# Characterization of Carcinogenic and Non-Carcinogenic Metal(loid)s in Water Within a Uranium-Mining-Impacted Region in Northwestern New Mexico, USA

**DOI:** 10.3390/ijerph23060800

**Published:** 2026-06-15

**Authors:** Christine Samuel-Nakamura, Abdul-Mehdi S. Ali

**Affiliations:** 1 School of Nursing, University of California, Los Angeles (UCLA), 4-246 Factor Bldg., Los Angeles, CA 90095, USA; 2 Department of Earth and Planetary Sciences, University of New Mexico, Northrop Hall MSCO 3-2040, Albuquerque, NM 87131, USA; mehdiali@unm.edu

**Keywords:** water, Navajo, arsenic, cadmium, uranium, mining, American Indian, carcinogen, cancer, environmental justice

## Abstract

**Highlights:**

**Public health relevance—How does this work relate to a public health issue?**

- Legacy uranium mining has contributed to environmental contamination concerns in American Indian communities where drinking water may serve as a pathway of exposure to carcinogenic and non-carcinogenic metal(loid)s.
- Environmental exposures to arsenic, cadmium, lead, uranium, and associated metal(loid)s are important public health concerns as several of these substances (As, Cd, Pb) have established links to cancer and other chronic diseases.

**Public health significance—Why is this work of significance to public health?**

- The study identified cadmium concentrations exceeding federal drinking water standards, providing evidence of ongoing environmental exposure to a known human carcinogen in a uranium-mining-impacted region.
- These findings contribute to understanding how legacy mining contamination may influence cumulative environmental exposures that could contribute to cancer and other long-term chronic disease risks in impacted communities.

**Public health implications—What are the key implications or messages for practitioners, policy makers and/or researchers in public health?**

- Continued investment in safe drinking water infrastructure, environmental remediation, and exposure-reduction strategies are needed to address environmental health and potential cancer risks associated with legacy mining contamination.
- Future longitudinal studies should focus on examining cumulative risk exposures to carcinogenic metal(loid) mixtures and evaluate their associations with cancer incidence, survivorship, and other long-term health outcomes in affected populations.

**Abstract:**

More than 500 unreclaimed mines and about 1100 associated waste sites remain on the Navajo Nation as a result of uranium (U) mining. This study evaluated the impact of U-mining water contamination in a region of Northwestern New Mexico. The goal of this study was to determine historical baseline concentrations of selected metal(loid)s: those found to be highly associated with cancer (arsenic (As), cadmium (Cd), lead (Pb)) and other associated metals: cesium (Cs), molybdenum (Mo), selenium (Se), thorium (Th), U, and vanadium (V), using inductively coupled plasma-mass spectrometry. Cadmium drinking water concentrations (10.64 μg/L) exceeded the United States Environmental Protection Agency Maximum Contaminant Levels (MCLs) of 5 μg/L. Overall, water mean concentration levels were 11.04 μg/L of Pb, 4.21 μg/L of As, 3.53 μg/L of U, 278.67 μg/L for Mo, 21.70 μg/L for V, 2.39 μg/L for Cs, and 7.75 μg/L for Se. These findings underscore the importance of improving access to safer water sources and highlight the need for continued environmental monitoring and research on exposure pathways associated with carcinogenicity and other negative health outcomes.

## 1. Introduction

Metal(loid) contamination may occur in uranium (U)-mining-impacted areas [1,2,3,4], where metals can migrate into the drinking water sources from anthropogenic sources. This is not the focus of this paper; however, the uranium and associated metal(loid)s were found to undergo rapid dissolution in one study [1]; a similar study found uranium to be mobile or migrate with precipitation and flood events [5]. The U-mining history and legacy of waste has been extensive and prolonged on Navajo or Diné lands; the extensive mining began in the 1940s and continued throughout the 1980s. A U-mining moratorium ban on Diné lands was initiated on 29 April 2005 [6]. A portion of this study also examined locally raised, harvested, and collected subsistence foods and medicinal plants [7] that have been reported elsewhere [3,4,8,9]. The findings of this current study focused on reporting exclusively on drinking water, several local studies have documented potential and active contamination of water sources within known intense U-mining-impacted areas [2,3,4]. The most common route of exposure to As was via water contamination [10].

Drinking water represents a critical pathway for exposure to hazardous metal(loid)s, especially in communities that rely on unregulated or untreated sources. This is a water- and food-insecure area and has been documented in several community studies that demonstrate a higher risk for water contamination concerns; a situation that also became more apparent during the COVID-19 pandemic [11,12]. In many Indigenous and rural communities, water is not only essential for consumption but also plays a central role in food preparation, agriculture and cultural practices. Participating in subsistence farming is considered a sacred role in this community and is richly steeped in its history and stories [13]. Water is part of the food web in terms of supporting traditional Diné foods (including medicinal plants and teas), is strongly rooted in identity and spirituality, and is connected in every aspect to foodways, whether it includes their cultivation, harvesting/collection, preparation, and contribution to cultural significance (e.g., for ceremonial activities such as cleansings and sweat lodges) [13,14]. Limited access to regulated water infrastructure, combined with reliance on hauled or locally sourced water can exacerbate exposure risks and contribute to broader environmental health disparities. This was a significant environmental health risk for this community, as well as a large number of AI communities that have been found to be in close proximity to more than 160,000 abandoned mining areas in the Western US alone [15]; these risks have direct detrimental effects and impacts on drinking water, food, and other important traditional cultural resources [2,16,17,18,19,20] and livelihoods [3,4,8,9].

Excessive metal(loid) exposures are of concern in chronic water ingestion and have various long-term health impacts, particularly cancer. Arsenic has been shown to cause cancer (CA) in humans (Group 1) according to the International Agency for Research on Cancer [21]; specifically, it has demonstrated mutagenic changes in human cells [22]. The primary source of contamination and exposure for humans was via drinking water [23]. It is uncommon to get As from food unless you eat a lot of high-As-containing foods (i.e., rice) [23]. Cadmium is associated with lung, kidney, prostate cancers and breast CA risk as documented by IARC (Group 1) [21,22,23,24,25]. Lead has also been shown to be a probable carcinogen to humans (Group 2A for bladder, lung and kidney CAs) [26]. This community has high rates of liver, myeloma, gallbladder, stomach and renal CAs [27]. Other studies have reported worsened outcomes for prostate [28] and breast CAs in American Indian groups [29]. These associations require further exploration. Arsenic, Cd, and Pb are known environmental toxins and are known carcinogens—how they interact with each other needs further investigation [22].

A number of metal(loid)s commonly associated with U mining have been liked to adverse health outcomes. Arsenic (particularly the inorganic form [30]), cadmium [31], and lead [32] are well-established toxicants with documented associations to carcinogenic and non-carcinogenic effects (i.e., bone accumulation). Water exposure risk is particularly high for inorganic As (iAs) [30,33]. As is a known contributor to untoward cardiovascular effects and diabetes [22]. Uranium exhibits both chemical and radiological toxicity [23], with evidence of renal [34] and skeletal impacts [35,36]. Natural U has been shown to co-occur with other metal(loid)s by virtue of decay series. Recent studies have shown an association between U and hypertension, as well as an increased risk for multiple chronic diseases, including diabetes and kidney disease [37,38,39]. Radium is part of the decay series of U and has been shown to be associated with lung cancer in miners [40]. In the literature, there are fewer studies reporting on water radium concentration levels in this community [41]. Other elements such as vanadium [42], selenium [43,44], molybdenum [45,46], cesium [47], and thorium [48] have been associated with a range of systemic effects at elevated exposure levels. Given the co-occurrence of these elements in mining-impacted environments, it is important to evaluate their presence collectively rather than in isolation. In addition to direct ingestion, environmental exposure pathways are complex and interconnected. In times past during non-drought conditions, community members relied primarily on natural precipitation for irrigation via “dry farming” [9,49]. Due to a decades-long drought, most families have relied more on direct application of local irrigation water. Community irrigation water access was a known farming challenge in this region [50,51] for numerous reasons. Water sources were primarily referred to as being “unregulated” [36,52]; examples of unregulated water sources included livestock wells, private wells, earthen dam water sources, and rainwater capture. Unregulated water is defined as those sources that are untreated and untested. Thus, their mitigation status is unknown, and they often do not meet primary drinking water standards set forth by the USEPA, states or tribes per the Safe Drinking Water Act [52]. A local study [2] reported that more than half of Diné participants drank from unregulated sources and >80% hauled water despite having public water or regulated water available in their homes. The network of exposures is numerous and complex. For example, the study area also contained mine waste [2] which may contribute to the leaching of contaminants into local water sources or may contribute to the deposition of windblown metal(loid)s onto soil, crops, plants, or open water sources. There was a risk of exposure to humans consuming livestock which have ingested contaminated water, crops or wild forage plants. Water insecurity factors were also of concern which included, the lack of indoor-plumbing [12], the need to travel great distances to obtain water, and the cost to travel to secure drinking water sources were significant challenges [2]. According to [5], the average cost of water for the metropolitan areas was $600 per acre-foot of water (1 acre-foot = 330,000 gallons of water), while for Diné community members the defrayed cost was about $43,000 per acre-foot of water [5]. The water costs were attributed to inaccessibility factors and transportation costs of water often over great distances. The average use of water by non-Diné US residents is about 190 gallons per day [5] while Diné people use about 10–100 gallons per day depending on water accessibility factors [53].

The present study goal aims to determine and characterize metals (Cd, Cs, Pb, Mo, Th, U, and V) and metal(loid) (As, Se) concentration levels in community drinking water samples on the Eastern Navajo Reservation in known high uranium-contamination areas as compared to other studies for the remaining parts of the reservation. This regional study attempted to: (a) address the gaps in knowledge by providing more detailed water ingestion and water use data (including a report on agricultural, medicinal and ceremonial uses), (b) provide historical baseline information on water use for certain metals (Cd, Pb) that have an established MCL, and (c) focused on, reporting and characterizing the metal(loid)s that according to the IARC are known to cause cancer. In summary, we wanted to report and characterize metal(loid)s and to provide a focus on those that are known to cause cancer in the current study area.

## 2. Materials and Methods

Water samples were collected from communities within a U-mining-impacted region of the Navajo Nation in Northwestern New Mexico and were analyzed for As, Cd, Cs, Pb, Mo, Se, Th, U, and V Sampling locations were found to be situated in close proximity to mine features, with the majority of the water samples (87.5%) collected using a water sampling protocol from within a 3.2 km radius of abandoned U mines and structures from the Eastern Agency of the Navajo Nation (NN); 12.5% of the water samples were collected outside a 3.2 km radius of mines/structures. There were five management and governing agencies on the Diné Nation (Eastern, Western, Chinle, Fort Defiance, and Shiprock agencies). Participants were identified through existing cohort data (Diné Network for Environmental Health (DiNEH) [2]) and supplemented by community outreach (word-of-mouth, home visits, and via “chapter” or community tribal meetings and events).

### 2.1. Setting

**Figure 1:**
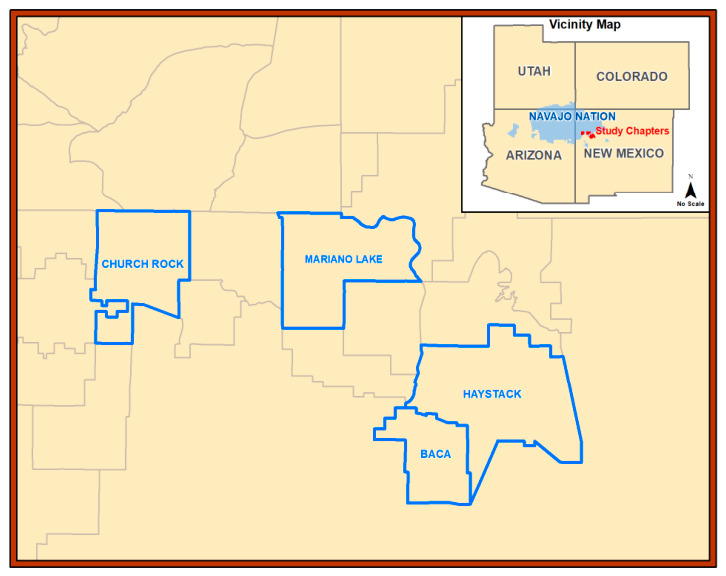
Map of research area in Northern New Mexico: Baca/Prewitt/Haystack, Churchrock, and Mariano Lake Chapter Communities of Diné Lands. The Navajo reservation is depicted in solid blue and spans Arizona, New Mexico and Utah.

The research site was in an arid-to-semi-arid region of the U.S. Southwest on Navajo lands. The average elevation was 2192 m. Four community chapters participated and provided water samples. Their combined community land mass was 1036 km^2^ (Figure 1). The average precipitation was less than 25 cm per year according to the climate meteorological data in New Mexico reported by the Western Regional Climate Center Western U.S. Climatic Historic Summaries (summary date range: January 2011–September 2012). In this community region the naturally occurring mineral deposits consisted of U and As [18].

The recruitment activities took place from May through September 2012. Water samples were collected during one harvest season from 8 August 2012 to 14 September 2012. The University of California, Los Angeles (UCLA) Institutional Review Board and the Navajo Nation Human Research Review Board (NNR-11.321) approved the study. Each adult study participant provided their written informed consent to participate in the study. Community and individual consents to participate in the study were obtained. This study contributes to a larger parent study, with results reported elsewhere for wild herbal tea [3], squash [9], medicinal plants [7], and the primary meat staple [4].

### 2.2. Human Water Use and Ingestion Questionnaire Data

A set of two questionnaires was utilized to gather harvester information. A general questionnaire, the Diné Plant–Animal–Human Questionnaire (DPAHQ) was used to collect participant demographic information and basic overall water and food use. The Diné Crop Intake Questionnaire (DCIQ) collected more detailed water use information, such as amount and frequency of water consumption and use within and outside the home, type and amount of water irrigation used on crops, medicinal plants, details of water use and storage, and those used for cooking, washing and bathing.

### 2.3. Water Sample Collection, Preservation, and Transport

Drinking water samples were collected from a variety of sources, including household and outdoor faucets or spigots via first-draw samples. Open and/or standing water samples from outdoor collection points and/or from multiple water containers were collected as composite water grab samples. Each 250 milliliter (mL) sample was placed in lab-grade, HM-analysis, PE containers, preserved with water bottles, nitric acid (HNO_3_), and stored on dry ice for transport. Water temperature and pH (Omega Engineering, 2012, Stamford, CT, USA) (HH12B) [Digital Probe] data were collected. Duplicate and blank samples were included for quality assurance.

### 2.4. Global Positioning System Data

Global Positioning System (GPS) instrumentation (Trimble Navigation Limited, Westminster, CO, USA) was utilized to document and analyze location information for all water samples and associated data. Field samples were marked and geocoded using a 2008 Trimble R GeoXT (Trimble Inc., Sunnyvale, CA, USA) [54]. Differential correction within 72 h of data capture was completed using GPS Pathfinder Office version 5.30 (Trimble Navigation Limited, Westminster, CO, USA).

### 2.5. Sample Analysis

The water samples were prepared and analyzed by the University of New Mexico Analytical Chemistry Laboratory, Earth and Planetary Sciences Department utilizing ICP-MS (PerkinElmer NexION 300D, Waltham, MA, USA). Sample analysis has been documented and reported in previous publications [3,4,7,9]. Aqueous samples were prepared by acid digestion protocol in which a 50 mL sample was transferred into digestion tubes. Five ml ultra-high purity (UHP) nitric acid (HNO_3_) and 2 mL hydrogen peroxide (H_2_O_2_) were added. Samples were heated gradually up to 95 °C. After digestion was completed, water samples were filtered using 0.45 micron filters then transferred into 50 mL volumic flasks and brought to volume using 18 mega ohm water. With each batch of samples, a reagent blank (5 mL HNO_3_ plus 2 mL H_2_O_2_) was digested and upon completion, the reagent blank sample was filtered then transferred into 50 mL volumetric flasks and brought to volume using 18 mega ohm water.

The method detection limits are U: 0.008 μg/L, Pb: 0.008 μg/L, Cd: 0.1 μg/L, and As: 0.3 μg/L. Three replicates of each sample were analyzed. Only a subset of metals were tested. The precision of the results demonstrated relative standard deviations varying from 7.1% to 13.8%.

### 2.6. Statistical Analysis

The statistical analysis software used was provided by SPSS 27 (IBM, Armonk, NY, USA) [55]. Descriptive statistics were used to summarize metal(loid) concentrations including means, ranges, and standard deviations. Metal(loid) water concentrations were reported in micrograms per liter (μg/L).

## 3. Results

### 3.1. Questionnaire Data: Human Water Ingestion and Use

Seventy-five percent of the participants (from eight households) reported not having access to a community or public water system. For crop irrigation water, the most frequently used water source was from rainwater capture (75%) and public or regulated water (25%). One study participant used public water exclusively to water crops. For livestock sheep watering the source that was most utilized was seasonal livestock dams (43%), followed by public regulated water (29%), and 14% equally between windmill water pump (intended for livestock use) and private well use (unregulated). The mean age of water consumers was 57 ± 10.9 years and they had lived in the current location for a mean of 51.50 ± 9.19 years. Sixty-three percent of study participants were women. On average, two water sources (comprising any combination of public water, rainwater capture/natural precipitation, and undetermined water sources) were utilized by each participant. While water users did not report actively drinking irrigation water currently, one participant did report consuming unregulated water that was intended for irrigation use in years past. The vast majority of irrigation water was stored in plastic or metal vessels (some repurposed). Water was frequently transported to the home, crop or livestock areas from multiple sources (such as a private wells, to and from the home, public water systems, from rainwater sources).

### 3.2. Metal(loid) Concentrations in Drinking Water

**Figure 2:**
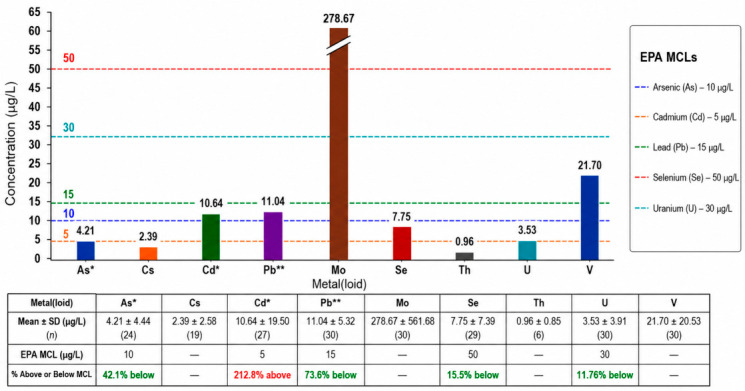
Metal(oid) concentrations in drinking water and associated Federal and Tribal EPA Maximum Concentration Levels (As, Cd, Pb, Se, & U). Note: No established EPA MCL (Cs, Mo, Th, & V); * International Agency for Research on Cancer (CA) Group 1: Carcinogenic to Humans or ** 2A: Probably Carcinogenic to Humans.

Of all mean water concentration levels, one out of nine metal(loid)s, Cd (10.64 ± 19.50 μg/L) exceeded the USEPA Maximum Contaminant Level (MCL; Figure 2) by 213% (Cd). Arsenic, Se, Pb and U concentrations were below the MCLs [56,57]. The MCL for Se is 50 μg/L [56,57]. Other metals were determined and reported elsewhere and were not covered under the established MCLs (Cs, Mo, Th and V). The mean water pH was neutral at 7.87 (SD = 0.69) and the mean temperature at sampling was 15.11 °C (SD = 12.7).

## 4. Discussion

### 4.1. Summary of Findings

The MCLs were exceeded for Cd (10.64 ± 19.50 μg/L) by more than 213% in study water samples [56,57]. The remainder of the metal(loid)s that have a water standard (As, Pb, Se, and U) did not exceed water MCLs [56,57,58]. Currently, USEPA MCLs do not exist for various metal exposures such as Cs, Mo, Th, and V. The pH of the water was within the EPA’s non-enforceable Secondary Drinking Water Regulations, which is recommended at an optimal pH range of 6.5 to 8.5 (current study mean 7.87 ± 0.70.) [57]. According to the harvester questionnaire water data, the metal concentration levels for one participant that utilized repurposed metal irrigation vessels contained the highest Cd concentrations and elevated Cs, Pb, and Mo concentrations in water compared to those that did not use repurposed metal tanks. The repurposed metal tanks were obtained from the Fort Wingate Army depot, a known contaminated munitions site [59]. At the time of sample collection, all study participants were informed of the importance of exclusively using food-grade water vessels for crop irrigation and were discouraged from consuming water from metal, non-food-grade water containers or those vessels of unknown origin or unpronounceable labeling, information comparable to recommendations of the NNEPA [60]. Agricultural water access remains a major issue [50,51], and residents may be pressed to use available water sources, which are often unregulated. Even though participants did not report actively drinking the irrigation water, it is still a cause for concern as it may be used for drinking, bathing, cooking, and water for locally consumed livestock. There was plant uptake of metal(loid)s through phytoaccumulation and translocation within the plants [3,7,9]. There was also livestock consumption of contaminated plants and metal(loid) accumulation in their various organs [4]. In the same study communities, deLemos [2] reported that 100% of study participants hauled (transported) water despite having a home public water source. Up to 30% of Diné homes lack access to regulated water. The lack of water infrastructure, history of extensive U mining, and co-occurrence of other metal(loid)s with U continue to be perceived as major health issues that need to be addressed on the Navajo reservation [18].

### 4.2. Other Regional and International Comparative Studies

Other local water quality studies on the New Mexico side of the NN undertaken by Erdei et al. [52] found that As was above the drinking water standard (13.6 ug/L), of nine water sources: two sources exceeded the MCL for As, four for U and three for Radium (Ra). Data regions from Arizona and Utah on the NN [61] found concentrations above the USEPA MCL for As and U. In the same area of the NN, Ingram and associates [5] found MCL exceedances for six metals and they consisted of As, U, V, Manganese (Mn), Lithium (Li), and calcium (Ca). Across five NN regulatory agencies, As (15% of 463 samples) and U (12.5% of 463 samples) exceedances were also reported by Hoover et al. [18]. Similar to this study, Credo et al. [61] reported that one water source had a Cd concentration exceedance; however, there were no Pb concentration exceedances; the other above local studies (excluding Credo et al. [61] did not determine Cd or Pb water concentrations in their study. Cd exceedances are of particular interest because it is an established Group 1 human carcinogen according to IARC; further determination and monitoring is needed for future studies and to address an important research gap. Similar to the Credo et al. [61] study, this study showed a water Cd MCL exceedance; however, unlike the other local studies, there were no exceedances found in water As or U. The current study sample size may have been too small to show sample exceedances for As or U.

Several international studies were found to be similar to the mining and waste type and extent or legacy of the current study area and they will be presented next with reports of similar metal(loid) concentration levels. A German study by Bister et al. [62] found post-U mining that Pb (120 μg/L) and U (380 μg/L) concentration levels persisted in ground water and river systems; this is similar to the current study. In Romania, Murarescu et al. [63], found elevated metal concentrations in ground water and surface water in a U-mining legacy area full of mining and tailings dump sites. They reported both groundwater and surface water concentrations, respectively: Cd 0.15–28.43 μg/L, 0.01–16.71 μg/L; Pb 3.62–162.33 μg/L, 1.25–28.02 μg/L. In a study in China, Yi et al. [64] demonstrated that U (3.33 μg/L) and Th (2.23 μg/L) exceeded background concentration water levels; pollution indices found high ecological risk in this region post-U mining.

### 4.3. Uranium and Other Associated Metal(loid)s

Overall, U concentrations in water samples were low and the lowest in each category. However, in U-mining-impacted areas, the importance of determining other co-metal(loids) and contaminants was as important as focusing on the primary metal mined. Other associated metals need further exploration. For example, a recent sole study by Erdei et al. [41] determined and reported radium levels and found MCL exceedances of 5 picocuries (pCi) per liter in three water sources, as well as four water sources exceeding the U MCL. Radium (separate from Radon, mentioned earlier) is a known human carcinogen and a constituent of U-mining ore [65]. The current study did not determine Ra levels but should be included in future samples to examine potential contribution and association to various CAs. In another regional study by Hoover et al. [18] they emphasized the risk of co-exposures to multiple contaminants in U impacted areas (specifically As and U). Determining the interactions between contaminants and the cumulative adverse health risks of multiple contaminants is needed. More research is needed to determine and evaluate co-contaminant exposures, allowing for improved characterization of health impacts.

### 4.4. Questionnaire Data: Drinking Water, Food Consumption, Other Uses

According to participant questionnaire data, the mean number of years of water ingestion was 17.50 ± 19.09 years. The amount of time residing within the water source area was 51.50 ± 9.19 years. The length of exposures related to participating in agricultural activities was extensive among this cohort. Exposure to metal(loid)s can occur during various stages of agricultural irrigation activities (including planting, cultivating, harvesting), food preparation, cooking, and washing. One study reported that food processing or cooking (via boiling, grilling, or baking) may alter Se levels, though research has yet to determine in which direction [66]. Another study found that As dissociates in water at a range of temperatures (via boiling) [67]. Determining the level and extent of exposure at various stages of water collection and storage along with the impact of food preparation and cooking is an area of future investigation.

### 4.5. Current Water Status and the Future

Exposure to unregulated irrigation water is still a major concern and should be avoided for use in drinking, food preparation and cooking. Food-grade water vessels should be used exclusively for drinking and any connection with the food chain. Whether there is an increased exposure risk from bathing and cooking needs examination through further research. It would be ideal to study exposure risk attributed to all water source uses in conjunction with the various ways foods (subsistence, non-subsistence, etc.) are grown, cleansed, prepared, cooked, and stored in order to further establish the health impacts of co-occurring contaminants. Tailored research is also suggested to examine the exposure of metal(loid)s to specific subpopulations, such as the very young, those of advanced age, those that are or may become pregnant/lactating women or those with chronic health conditions, particularly cancer, diabetes, renal or liver disease or failure, cardiovascular disease, altered immune function/autoimmune disorders. The U-mining era spans more than eight decades in this region and its contribution to the cancer latency period has matured. More work can be undertaken to examine and report on long-term metal(loid) exposures (even exposures at low concentration levels) and their association with various cancers.

### 4.6. Limitations

Water from the study areas may have been transported or collected from multiple sources, perhaps even out of the designated sampling areas (>3.2 km radius of mines/structures). Transported water sources may be more or less contaminated than the home sampling areas. In addition, water metal(loid) concentrations are known to change temporally or seasonally. Thus, our samples provided concentration levels at only one point in time. Climate change and drought impacts are factors that have not been explored or well-studied. Some reports suggest that drought conditions may contribute to more concentrated contaminants [68]. Hence, resampling water sources during drought (and for the future) is advisable. Thus, future research should strive to incorporate longitudinal and multi-seasonal examinations. Reevaluation in series or over multiple seasons would be beneficial and provide an improved understanding of the variations attributed to climate change, seasonal (e.g., runoff, windy seasons) or climatic factors (i.e., drought, flood). Another area of exploration should include air monitoring studies which ideally would provide insight into the extent and amount of metal(loid)s settling on open water sources via aerosolization.

## 5. Conclusions

The study showed that Cd concentration levels exceeded the USEPA MCL. Arsenic, U, Pb, and Se concentration levels did not exceed the MCLs. In most instances, U concentration levels were predominately low in the majority of the samples; nevertheless, there have been elevated concentrations of co-metals in water in this and local studies and their continued monitoring and surveillance is needed for understanding and addressing their cumulative and mixture exposures. Unregulated water use and ingestion are still problems in this community where mixed-metal mining continues to exist extensively. More research is needed for metal(loid)s that promote carcinogenicity and other long-term health sequelae.

## 6. Recommendations

- Continue long-term environmental monitoring and surveillance of water, soil, and food sources in uranium (U)-impacted regions.
- Encourage continued use of water and soil mapping tools in high-risk areas as previously recommended by researchers [2].
- Promote water education resources and outreach provided by the Tribe EPA [59] and other resources.
- Continue implementing food study recommendations related to environmental contaminants and exposure pathways [3,4].
- Expand future studies to include human biological samples to better link water and food exposures with metal(loid) contamination and long-term health outcomes, specifically cancer.
- Increase American Indian/Alaska Native (AI/AN) participation in cancer research studies and clinical trials to address longstanding underrepresentation and disparities in cancer research [67,68].
- Research efforts should include training AI/AN researchers, students (pre-K to postdoc), staff, tribal community members, include building local capacity and focus on building research benefits to the Tribe.
- Explore the use of big data and population-level cancer databases to address gaps in cancer surveillance and outcomes across the cancer care continuum in AI/AN populations.
- Maintain and strengthen Diné Nation involvement in all aspects of environmental health research, including study development, implementation, interpretation, and dissemination.
- Support continued enforcement and development of Diné Nation uranium mining moratorium policies and associated environmental health policies and protections.
- Address the finding that cadmium (Cd) concentrations exceeded the Maximum Contaminant Level (MCL), emphasizing that ingestion of unregulated water remains a major public health concern.
- Continue recommending avoidance of non-food-grade water vessels and open water sources (e.g., livestock-use sources, ephemeral water, or rainwater collection sources) for drinking and food-chain use per Tribal EPA recommendations [59].
- Create feasible avenues that improve (and test) access to clean, safe, and regulated drinking water supplies in impacted communities [18].
- Develop cumulative risk assessment tools capable of evaluating combined exposures to multiple co-contaminants rather than single-contaminant approaches.
- Include additional carcinogenic metals and radionuclides (e.g., Cd and Ra) in future environmental and health studies to address existing research gaps.
- Continue monitoring for carcinogenicity and other long-term health impacts associated with chronic low-level metal(loid) exposure.
- Recognize and address the complex interaction between environmental, socioeconomic, historical, and cultural factors that contribute to cancer disparities and outcomes.
- Address Social Determinants of Health (SDH) that affect the cancer care continuum, including prevention, screening, diagnosis, treatment, survivorship, and palliative care, particularly in rural, remote, and hard-to-reach or hidden communities [69,70].
- Improve access to safe water, food security, nutrition, transportation, and culturally relevant resources needed to support cancer care and overall health.
- Encourage additional research examining sociocultural, historical factors, and structural racism within SDH frameworks for AI/AN communities [68].
- Involve community members, leadership, and health policy makers in developing solutions related to cancer care accessibility, environmental health, water security, and food security.
- Recognize that water and food insecurity are likely to worsen with climate change and require proactive intervention strategies [12,71].
- Support restoration and integration of Traditional Ecological Knowledge (TEK) principles and methodologies in environmental health research and policy development [8,72].
- Develop environmental health policies that more comprehensively address environmental contamination, health disparities, environmental justice, and social justice concerns in American Indian and Indigenous communities.
- Ensure that environmental policy development, management, and implementation occur through full and equitable partnerships with sovereign Tribal Nations.

## Data Availability

The data generated and analyzed in this study fall under Navajo Nation Data Sovereignty rights and are not publicly available; they are under the ownership of the Navajo Nation.

## Abbreviations

The following abbreviations are used in this manuscript:

U	Uranium
As	Arsenic
Cd	Cadmium
Pb	Lead
Cs	Cesium
Mo	Molybdenum
Se	Selenium
Th	Thorium
V	Vanadium
MCL	Maximum Contaminant Level
AI/AN	American Indian/Alaska Native
CA	Cancer
IARC	International Agency for Research on Cancer
iAs	Inorganic Arsenic
USEPA	United States Environmental Protection Agency
NN	Navajo Nation
DPAHQ	Diné Plant–Animal–Human Questionnaire
DCIQ	Diné Crop Intake Questionnaire
mL	Milliliter
HNO_3_	Nitric acid
GPS	Global Positioning System
GIS	Geographic Information Systems
µg/L	Micrograms per liter
Ra	Radium
pCi/L	Picocuries per liter
SDH	Social Determinants of Health
TEK	Traditional Ecological Knowledge
